# Proximal Olecranon Free Flap for Cystic Scaphoid Nonunion: An Anatomical Feasibility Study

**DOI:** 10.1177/15589447251329569

**Published:** 2025-04-23

**Authors:** Laura C. Burlage, Liron Duraku, Tim Wang, Brahman Shankar Sivakumar

**Affiliations:** 1 Amsterdam University Medical Center, The Netherlands; 2 Royal North Shore Hospital, St Leonards, NSW, Australia; 3 Sydney Adventist Hospital, Wahroonga, NSW, Australia; 4 Hornsby Ku-ring-gai Hospital, NSW, Australia; 5 Nepean Hospital, Kingswood, NSW, Australia; 6 Australian Research Collaboration on Hands (ARCH), Mudgeeraba, QLD, Australia; 7 The University of Sydney, Camperdown, NSW, Australia

**Keywords:** vascularized bone graft, upper extremity, scaphoid union, microsurgery, bone reconstruction

## Abstract

**Background:**

The treatment of scaphoid nonunion is challenging, with one approach aiming to provide vascularized bone to encourage union. While the iliac crest and medial femoral condyle are well-described donor sites for osseous flaps, they require violation of a separate limb and confer particular donor site morbidities. We investigate the viability of using a proximal olecranon osseous free flap in the setting of scaphoid nonunion.

**Methods:**

Ten proximal olecranon free flaps were harvested in cadaveric specimens, and the length of the pedicle, diameter of the pedicle, number of perforators and quality of bone graft harvested were recorded. Furthermore, a volar approach to the scaphoid was performed, and the shortest distance from the scaphoid to the radial artery noted, to determine whether utilization of the olecranon free flap was possible without grafting.

**Results:**

The posterior ulnar recurrent artery [PURA] was present in all specimens. The median pedicle length from take-off of the PURA to the olecranon flap was 65 (62.2-71.0) mm. The number of visible periosteal perforators varied between 1 and 2 per specimen. The median diameter of the main perforator before dividing into subperiosteal branches was 2 (2.1-2.5) mm. The quality of the bone graft harvested was mainly assessed as good (n = 5) or moderate (n = 4). The mean shortest distance from scaphoid to radial artery was 10 mm.

**Conclusions:**

The olecranon free flap is a suitable alternative source of vascularized bone for scaphoid nonunion.

## Introduction

The scaphoid is the most commonly fractured carpal bone, accounting for up to 80% of all carpal fracture.^1^ While most scaphoid fractures will heal with nonoperative management, nonunion is noted in 10% to 15% of patients.^2^ Patient factors (such as smoking, fracture comminution and location, and delays to diagnosis and treatment) predispose the scaphoid to nonunion, as do anatomical factors, including a high proportion of cartilage cover and a retrograde blood supply impairing perfusion to the proximal pole in the setting of fracture (indicative of the bone’s perilous vascularity). Untreated scaphoid nonunion may lead to alterations in wrist kinematics, secondary arthrosis, and long-term pain necessitating further treatment.

Although the general principle in the management of cystic nonunion of the scaphoid remains adequate debridement, elimination of deformity and fixation with incorporation of autologous bone graft, divergent schools of thought exist on how best to achieve this.^3^ Arthroscopic debridement and percutaneous fixation espouses maintenance of the surrounding soft tissue—this serves to preserve vascularity and ligamentous attachments, and encourage union and normal wrist biomechanics.^4^ Nonvascularized bone can also be delivered through an open approach, with the use of a corticocancellous block yielding structural support to correct a humpback deformity. An alternate philosophy prioritizes the provision of vascularity, with incorporation of an osseous flap providing greater osteoconductive, osteoinductive, and osteogenic properties.^5^ The optimal source of vascularized bone when embarking on this route remains a source of conjecture.

Pedicled osseous flaps (such as those harvested from the volar aspect of the distal radius or the floor of the extensor compartments) may be technically challenging to harvest; require rotation around a pivot point that can impede positioning or compromise the pedicle; and can deliver inadequate bone to facilitate correction of humpback deformity.^6,7^ Free osseous flaps, which are commonly raised from the iliac crest or medial femoral condyle (MFC), require violation of a distant operative site or limb, and can be associated with morbidity distinct to each donor site.^8,9^

Yang et al. previously described an osseous free flap from the proximal olecranon, highlighting the vascular architecture and describing the anatomical parameters.^5^ Here, we build upon that work, concentrating on the flap’s utility in the setting of a cystic nonunion of the scaphoid.

## Methods

This cadaveric study was covered under blanket institutional approval. A sample of convenience of 10 cadaveric specimens, amputated proximal to the elbow, were used. All cadaveric dissections were performed by a single surgeon, with a separate clinician undertaking and recording measurements. Each cadaver was prepared and measured in the same setting to prevent tissue desiccation.

The proximal olecranon free flap is raised on the posterior ulnar recurrent artery (PURA), a branch of the ulnar artery proper.^4^ The ulnar artery arises from the bifurcation of the brachial artery at the cubital fossa and gives rise to the anterior ulnar recurrent artery initially, followed by the posterior ulnar recurrent branch. The PURA possesses a larger caliber then the anterior ulnar recurrent branch, and courses posteromedial, running between the flexor digitorum superficialis and profundus muscles to lie deep to the flexor carpi ulnaris [FCU] close to the ulnar nerve, which lies just volar and slightly superficial.^4,8^ Notably, it gives rise to a number of perforating branches to the medial surface of the olecranon and anastomoses with the superior ulnar recurrent and inferior ulnar collateral arteries to yield a medial arcade.^10,11^

**Figure 1.**
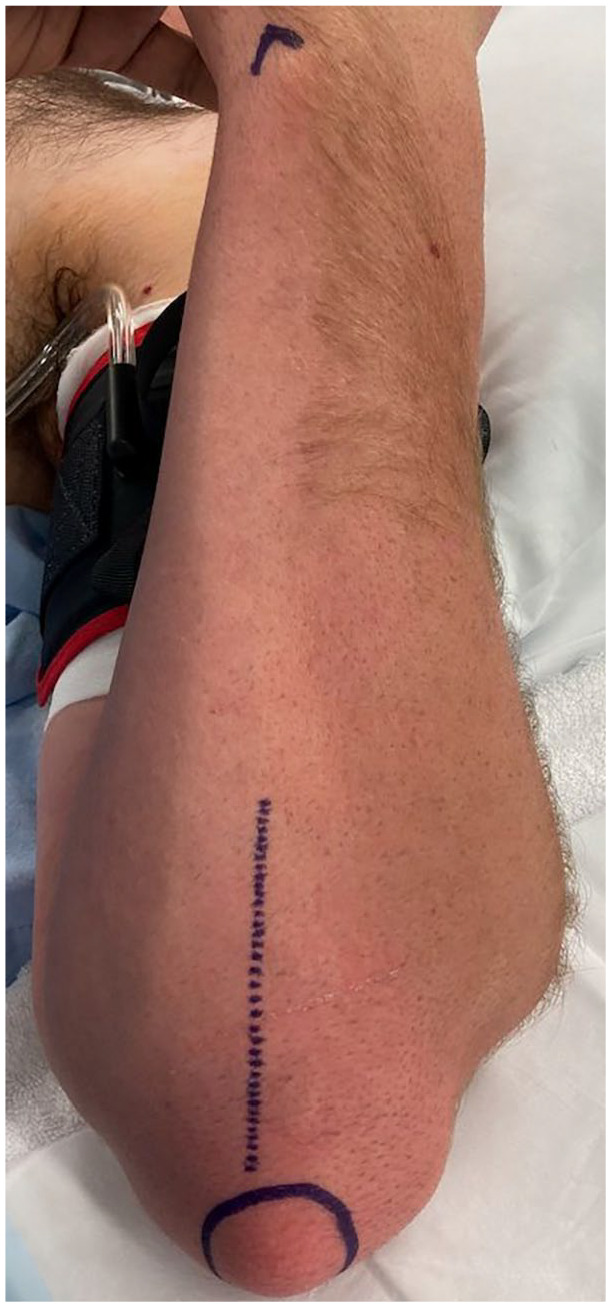
Skin marking for incision.

**Figure 2.**
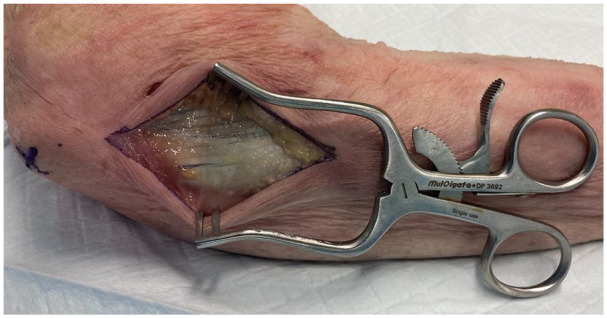
Fascia overlying flexor carpi ulnaris.

**Figure 3.**
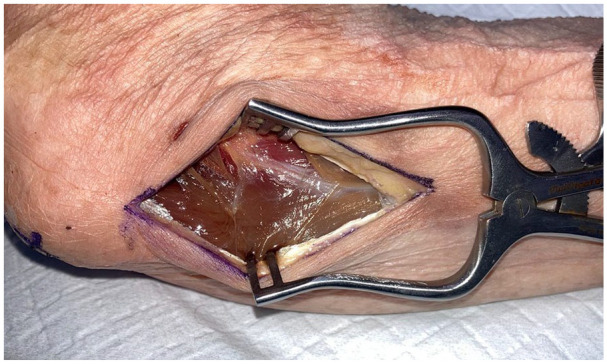
Fascia divided to expose flexor carpi ulnaris.

**Figure 4.**
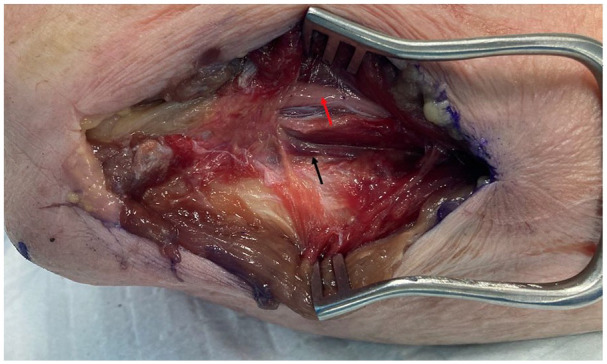
Flexor carpi ulnaris split and retracted to expose vascular pedicle of posterior ulnar recurrent artery (black arrow) and ulnar nerve (red arrow).

**Figure 5.**
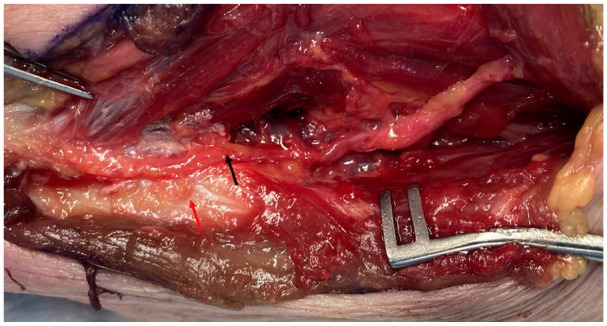
Pedicle dissected out (black arrow), with periosteal perforators on olecranon (red arrow). Ulnar nerve has been retracted anteriorly.

To raise these flaps, the cadaveric arms were positioned with the ulnar side of the elbow facing up, to simulate a supine patient with the shoulder abducted and externally rotated to present the operative site. The tip of the olecranon and the ulnar styloid were identified. An incision was performed 1 cm ulnar to the olecranon, extending from 1 cm distal to the olecranon tip for a length of approximately 10 cm (Figure 1). The subcutaneous fat and fascia overlying the FCU were divided, and the muscle fibers of the FCU muscle carefully split and blunt dissected from the olecranon and proximal ulna (Figures 2 and 3). The pedicle of the PURA, upon which this flap is based, was identified lying just deep to the muscle mass of the FCU (Figure 4). A number of muscular perforators arise from the PURA and were ligated; care was taken to identify and preserve all periosteal perforators. The ulnar nerve lies just superficial and volar to the PURA and was identified early to prevent iatrogenic damage—if necessary, it was released to obtain adequate exposure. Once the pedicle was isolated, it was dissected distally until its take-off from the ulnar artery (Figure 5).

**Figure 6.**
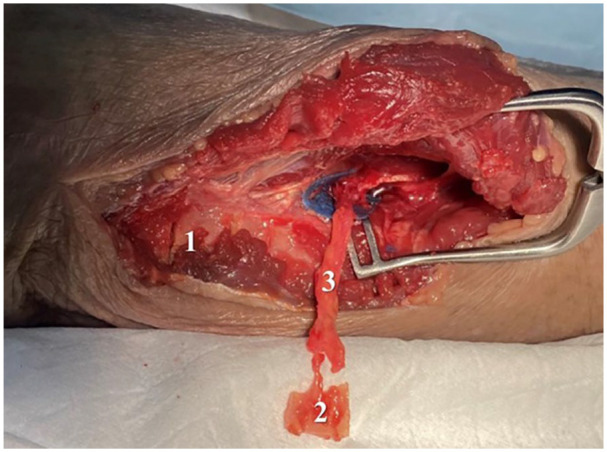
Osseous flap raised (2), with donor site (1) and posterior ulnar recurrent artery pedicle (3).

The osseous flap was then harvested using an oscillating saw and various osteotomies. Coronal plane osteotomies were created volar to the sublime tubercle and dorsal to the posterior border of the ulna; axial plane osteotomies were produced proximal and distal to the perforators, taking care to stay at least 2 cm distal to the tip of the olecranon to prevent fracture; and a sagittal plane osteotomy was generated by passing the saw from the posterior aspect of the ulna toward the coronoid process, approximately 1 cm radial to the ulnar border, to fashion the base of the osseous flap. The flap was extracted carefully using a stacked osteotome method and levering through the sagittal osteotomy (Figure 6). The cadaveric dissection was completed by performing a volar approach to the scaphoid, with the bone accessed via a longitudinal capsulotomy.

A number of measurements were then performed. The length of the pedicle from take-off to flap was assessed, as well as its diameter and the number of visible perforators it gave rise to. The quality of the bone raised was graded, with “good” allocated when a block of cancellous bone was present with a cortical shell, “moderate” assigned when the cancellous bone was partially fragmented, and “poor” ascribed when only a cortical shell was harvested. The shortest distance from the radial artery to the mid-point of the scaphoid was also measured, to replicate the minimal pedicle length required if the flap were to be used for reconstruction of a scaphoid waist defect.

Due to the small sample size, quantitative data were evaluated through medians with interquartile range.^12^

## Results

The 10 cadaveric specimens were donated by 8 males and 2 females, with a median age of 79 (76.3-84.8) years (Table 1). The PURA and medial arcade were present in all specimens. The ulnar nerve was only released in 3 of 10 specimens. The median pedicle length from take-off of the PURA to the olecranon flap was 65 (62.2-71.0) mm. The number of visible periosteal perforators varied between 1 and 2 per specimen. The median diameter of the main perforator before dividing into subperiosteal branches was 2 (2.1-2.5) mm. The quality of the bone graft harvested was mainly assessed as good (n = 5) or moderate (n = 4). In terms of the recipient site, the shortest distance from the mid-point of the scaphoid to the radial artery was 10 (8.5-12.1) mm.

**Table 1.** Cadaveric Measurements Data.

Cadaveric variables	Number (percentage) or median (interquartile range
Male sex—number (%)	8 (80%)
Age	79 (76.3-84.8)
Length pedicle (mm)	65 (62.2-71.0)
Number of perforators visible	1 (1-1.8)
Diameter of main perforator before division into periosteal branches (mm)	2 (2.1-2.5)
Shortest distance from scaphoid to radial artery (mm)	10 (8.5-12.1)
Necessity of release ulnar nerve—number (%)	3 (33%)
Quality of the graft bone harvested—number (%)	
- Good	4 (44%)
- Moderate	4 (44%)
- Poor	1 (11%)

*Note.* mm = millimeters.

* Data are presented as median with interquartile range, if not specified otherwise.

## Discussions

Free osseous flaps for use in the reconstruction of cystic nonunion in the scaphoid have traditionally been harvested from the MFC or iliac crest.^13,14^ However, harvesting either of these flap requires violation of a distant surgical site and is associated with specific donor site morbidity. Harvesting bone from the iliac crest may be associated with damage to the lateral femoral cutaneous nerve, fracture, and long-term pain limiting mobility;^15,16^ meanwhile, raising an MFC flap can result in saphenous nerve injury, femoral fracture, and ongoing knee pain.^17,18^

The olecranon has been routinely used as a source of nonvascularized bone graft and is particularly useful for upper limb surgery due to its proximity. The vascular anatomy of this region has been more intimately elucidated in recent years, leading to the conception of the olecranon free flap.^4,11^ The proximal olecranon free flap is an alternative source of vascularized bone, and offers a number of advantages for use in the upper limb, including confinement of operative morbidity to a single limb; avoidance of weight-bearing structures that may limit mobility; and the possibility of a chimeric flap with incorporation of a portion of the FCU muscle if required.^4^

This flap can be considered in the setting of small critical osseous defects in the upper extremity, such as a cystic nonunion of the scaphoid. Contra-indications include a requirement for larger osseous reconstitution (such as reconstruction of the distal radius following excision of a giant cell tumor) or prior trauma resulting in damage to the vascular supply of the proximal ulna. Care must be taken during the approach to avoid damage to the posterior branch of the medial antebrachial cutaneous nerve, which can be avoided by biasing the incision dorsally in the line between the olecranon and ulnar styloid. Patients should be warned that dissection of the FCU is necessary and may contribute to postoperative pain and mild weakness in wrist flexion—other donor site morbidity may include hematoma, fracture, and infection. The ulnar nerve runs close to the vascular pedicle and must be identified and protected, with a plan to neurolyse it if necessary to avoid traction. The most proximal axial plane osteotomy should be performed at least 2 to 3 cm distal to the tip of the olecranon to prevent fracture or damage to the triceps insertion.^4^ When insetting the flap, the pedicle must be arranged in a manner to prevent kinking and restriction of flow.

This study validates the findings of Yang et al in ascertaining a reliable pedicle with a median diameter of 2 mm and at least one periosteal perforator in all specimens.^5^ It also finds that the quality of bone harvested was moderate or good in 90% of specimens. Notably, the pedicle length of 65 mm was abundant when compared to the distance between the scaphoid and radial artery, which we envision would be its primary utility.

This study has limitations. The cadaveric donors were older, with likely osteoporotic bone, and replication in younger patients would be expected to yield a robust bony flap. Furthermore, the cadaveric tissue may have been altered by the process of preparation and preservation. Although the harvest was straightforward in this study, it is difficult to generalize due to the small cohort assessed, and dissection in a young muscular patient may prove more troublesome.

However, this study validates the work that has been previously performed and finds that utilization of a proximal olecranon free flap in the setting of scaphoid nonunion is a feasible option. Further clinical studies are needed to evaluate the effectiveness, safety, and postoperative outcomes of this technique *in vivo*.

## Conclusion

This study described a straightforward approach to harvesting a vascularized olecranon free flap and confirms consistent flap and pedicle measurements. Vascularized olecranon bone graft can therefore be considered as an interesting alternative vascularized bone graft in the setting of scaphoid nonunion.
